# Supplementary material for: An Increase in Fat Mass Index Predicts a Deterioration of Running Speed
Source: Nutrients. 2019 Mar 25;11(3):701. doi: 10.3390/nu11030701 (PMC6471649; doi:10.3390/nu11030701)

SUPPLEMENTAL MATERIAL

Table S1: Univariate linear mixed regression models for prediction of running speed

Table S2: Comparisons of multivariate linear mixed regression models

Figure S1: Flow-chart showing the selection of the included participants.

Figure S2: Predicted speed of each participant according to model 5 against age, according to the baseline body mass index [BMI; A], fat-free mass index [FMI; B] or fat mass index [FFMI; C]. Each body composition parameter was dichotomized, based on the median value at baseline. Median values for women and men were 21.9 and 24.1 kg/m^2 for BMI, 15.7 and 19.2 kg/m^2 for FFMI, and 6.3 and 4.9 kg/m^2 for FMI, respectively.

Supplemental table 1: Univariate linear mixed regression models for prediction of running speed in women [377 participants, 1419 observations] and men [509 participants, 2161 observations]

	Women				Men			
	ß	95% CI		p	ß	95% CI		p
Age [years]				<0.001				<0.001
15-24	0.00				0.00			
25-34	-0.23	-0.51	0.06		-0.17	-0.46	0.11	
35-44	-0.31	-0.63	0.01		-0.24	-0.55	0.08	
45-54	-0.61	-0.94	-0.28		-0.58	-0.91	-0.25	
55-64	-1.10	-1.45	-0.75		-1.50	-1.85	-1.15	
≥65	-1.92	-2.32	-1.52		-2.92	-3.31	-2.53	
Body mass index [kg/m^2]	-0.23	-0.27	-0.20	<0.001	-0.41	-0.45	-0.37	<0.001
Fat-free mass index [kg/m^2]	-0.07	-0.14	-0.01	0.059	-0.20	-0.26	-0.14	<0.001
Fat mass index [kg/m^2]	-0.34	-0.39	-0.29	<0.001	-0.50	-0.54	-0.45	<0.001
Year of measurement [yrs]				<0.001				<0.001
1999-2001	0.00				0.00			
2002-2004	-0.01	-0.12	0.12		-0.26	-0.38	-0.13	
2005-2007	-0.19	-0.32	-0.06		-0.48	-0.62	-0.35	
2008-2010	-0.27	-0.41	-0.12		-0.73	-0.87	-0.60	
2011-2013	-0.78	-0.93	-0.63		-1.43	-1.57	-1.28	
2014-2016	-0.81	-0.96	-0.64		-1.49	-1.64	-1.33	
Temperature [°C]	-0.01	-0.02	0.01	0.267	0.01	0.01	0.02	0.017
Relative humidity [%]	0.01	0.01	0.02	<0.001	0.02	0.02	0.03	<0.001

Supplemental table 2: Comparisons of linear mixed regression models

	Model 1	Model 2	Model 3	Model 4	Model 5
**Women [n=377, 1419 observations]**					
Bayesian information criterion	3618.8	3501.1[a]	3624.1	3450.9[a]	3458.1[a, b]
**Men [n=509, 2161 observations]**					
Bayesian information criterion	6136.2	5794.1[a]	6115.2[a]	5800.8[a]	5755.6 [a, b, c]

Model 1: categories of age, categories of year of measurement, temperature, relative humidity
Model 2: model 1 + body mass index
Model 3: model 1 + fat-free mass index
Model 4: model 1 + fat mass index
Model 5: model 1 + fat-free mass index + fat mass index
[a]p<0.001[likelihood-ratio test] vs. model 1
[b]p<0.001 [likelihood-ratio test] vs. model 3
[c]p<0.001 [likelihood-ratio test] vs. model 4

Supplemental figure 1

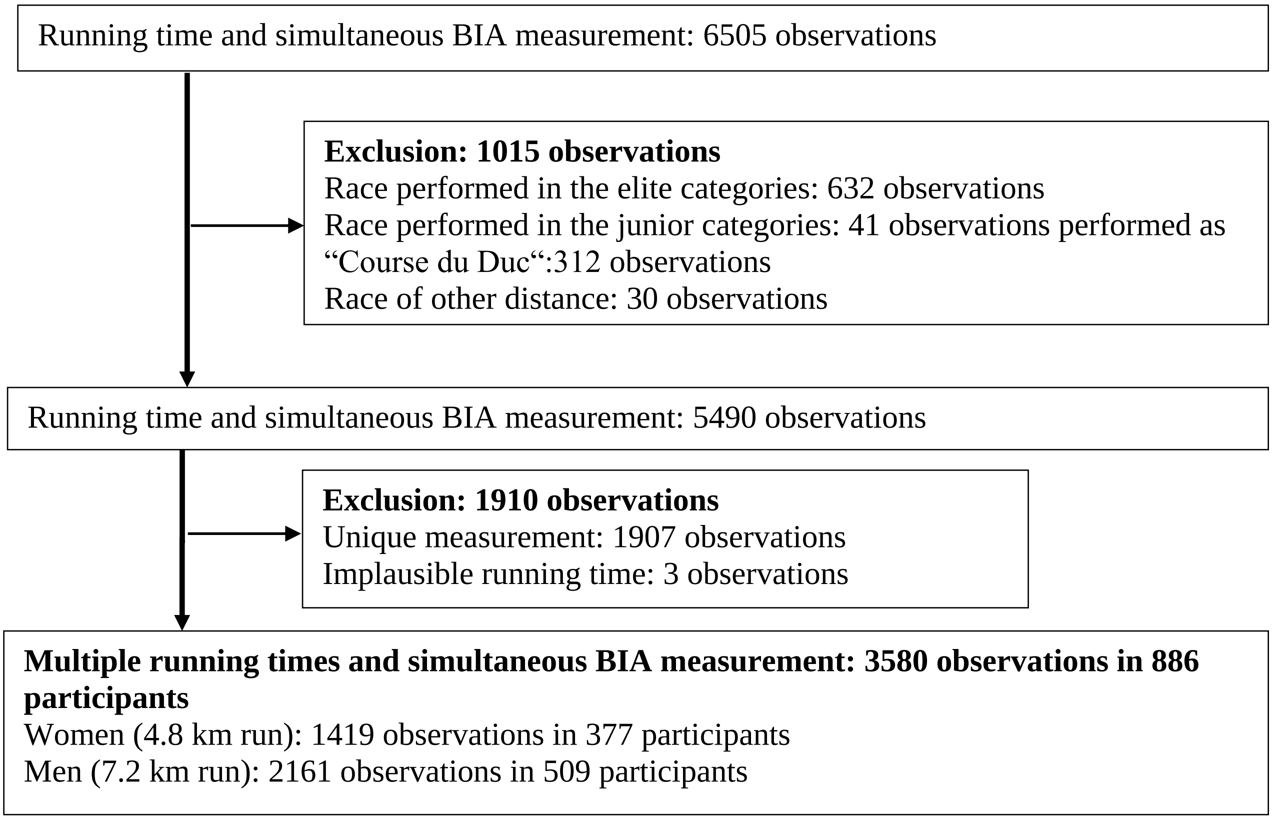

Supplemental figure 2

A.

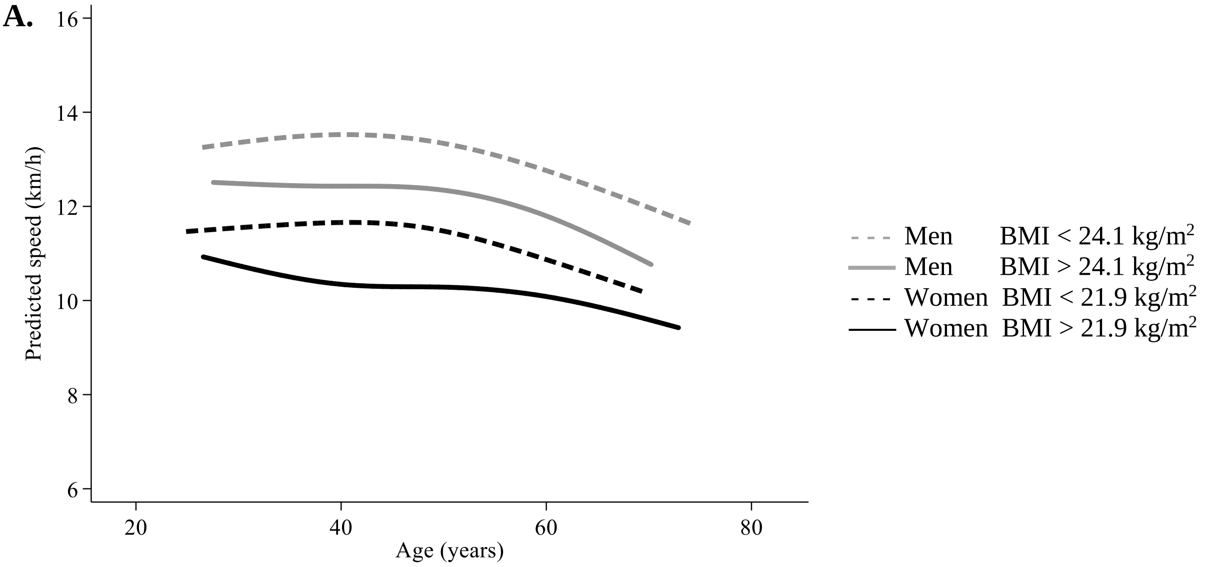

B.

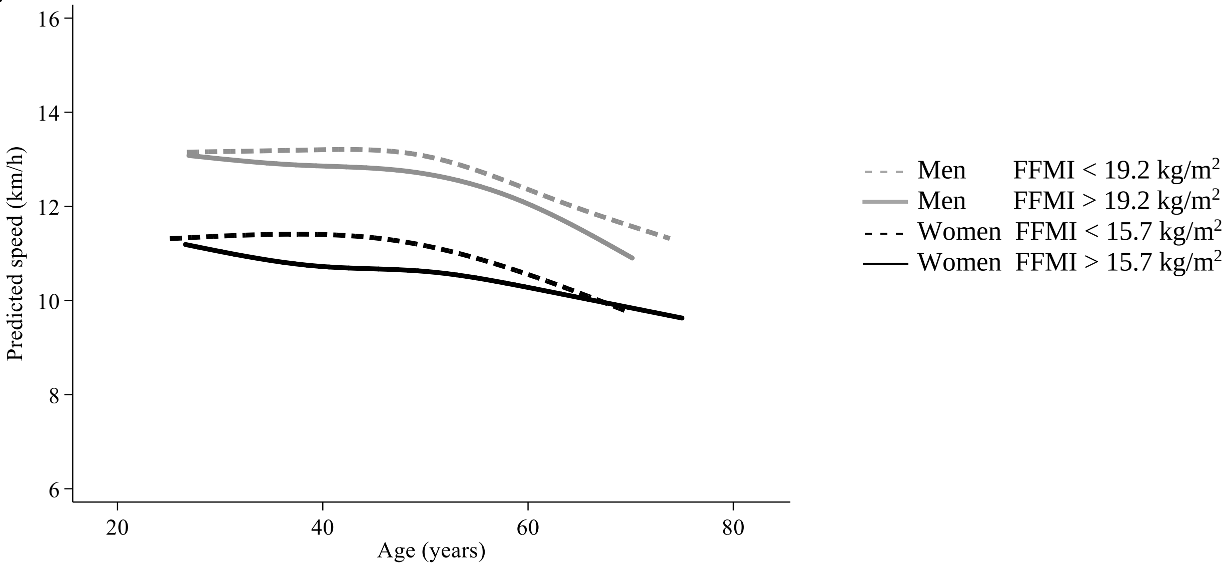

C.

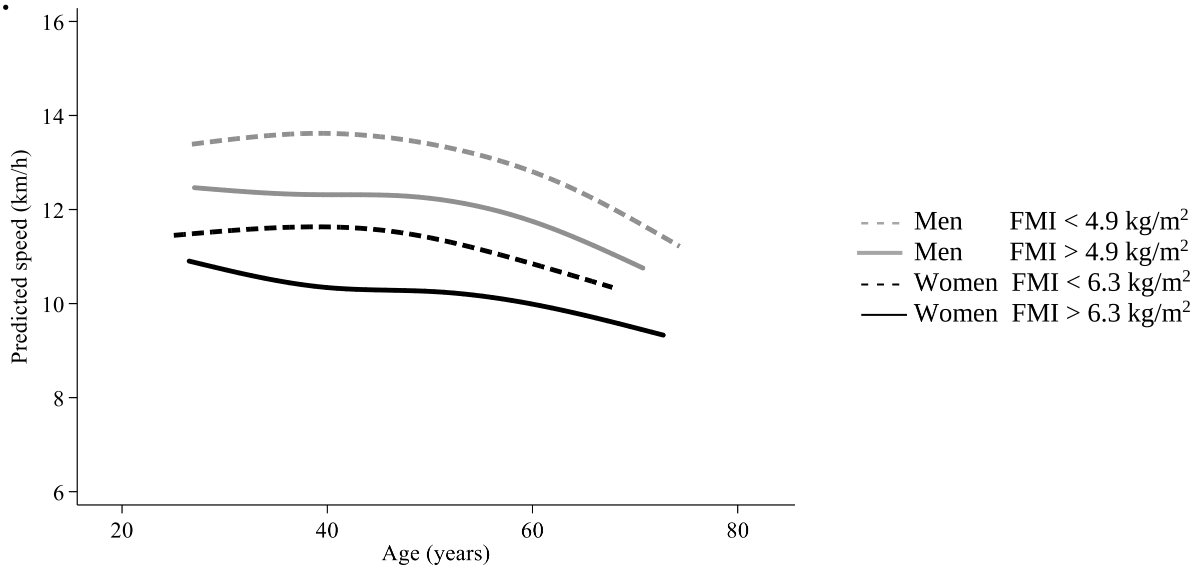

Supplement: Supplementary file 1 [file nutrients-11-00701-s001.pdf]
